# Have plants evolved to self-immolate?

**DOI:** 10.3389/fpls.2014.00590

**Published:** 2014-11-04

**Authors:** David M. J. S. Bowman, Ben J. French, Lynda D. Prior

**Affiliations:** School of Biological Sciences, University of TasmaniaHobart, TAS, Australia

**Keywords:** biomass burning, evolution, fire regime, landscape fire, niche construction, plant regeneration, plant traits

## Abstract

By definition fire prone ecosystems have highly combustible plants, leading to the hypothesis, first formally stated by Mutch in 1970, that community flammability is the product of natural selection of flammable traits. However, proving the “Mutch hypothesis” has presented an enormous challenge for fire ecologists given the difficulty in establishing cause and effect between landscape fire and flammable plant traits. Individual plant traits (such as leaf moisture content, retention of dead branches and foliage, oil rich foliage) are known to affect the flammability of plants but there is no evidence these characters evolved specifically to self-immolate, although some of these traits may have been secondarily modified to increase the propensity to burn. Demonstrating individual benefits from self-immolation is extraordinarily difficult, given the intersection of the physical environmental factors that control landscape fire (fuel production, dryness and ignitions) with community flammability properties that emerge from numerous traits of multiple species (canopy cover and litter bed bulk density). It is more parsimonious to conclude plants have evolved mechanisms to tolerate, but not promote, landscape fire.

## Introduction

The combination of carbon rich biomass, atmospheric oxygen, and ignitions makes landscape fire inevitable on Earth (Bowman et al., 2009). However, the occurrence, spread, and energy released by landscape fires is controlled by the physical environment. The most prominent environmental factor is climate because it influences the production of biomass, fuel arrangement across landscapes and its dryness, as well as providing lightning ignitions (Bradstock et al., 2012). The only life-forms that make fire are humans, and we, like our antecedents, are powerful agents in influencing the occurrence and spread of fires, given our capacities to modify fuels, provide ignitions and suppress fires (Bowman et al., 2011; Archibald et al., 2012). To what degree plant life has influenced the occurrence, extent and intensity of landscape fire remains controversial (Bradshaw et al., 2011a,b; Keeley et al., 2011b). Mutch (1970) hypothesized that “fire dependent plant communities burn more readily than non-fire dependent communities because natural selection has favored characteristics that make them more flammable” (Table 1). The “Mutch hypothesis” has logical appeal and is intellectually consequential for fire ecology and pyrogeography because it provides these disciplines with an evolutionary platform. However, because landscape fires affect entire plant communities rather than being restricted to individuals with heritable flammable characteristics, it is difficult to avoid group selection arguments (Snyder, 1984; Troumbis and Trabaud, 1989; Bond and Midgley, 1995; Scarff and Westoby, 2006).

**Table 1:** **Summary of hypotheses regarding evolution of flammable traits in plants, and possible examples**.

**Syndrome**	**Ancestral state**	**Evolved state**	**Example**	**References**
Mutch	Recovery/tolerance of fire	High flammability	*Eucalyptus*	Crisp et al., 2011
Mutch's converse	High flammability	Recovery/tolerance of fire	Serotiny and thick bark in *Pinus* Thick bark, xylopodia in savanna plants Fire-cued flowering in orchids	He et al., 2012 Simon et al., 2009 Bytebier et al., 2011
Midgley's alternative	High flammability	Low flammability	Branch shedding in *Pinus*	He et al., 2012

It is important to note that proving evolution of flammable traits, fire tolerance and post fire recovery demands extraordinarily rigorous studies that are yet to be achieved (Bradshaw et al., 2011a,b; Keeley et al., 2011b). We call this stricture “Bradshaw's null.”

A number of theoretical models have attempted to reconcile the evolution of flammability with individualistic selection theory by proposing ways that self-immolation can increase individual fitness or advantage to their offspring (Bond and Midgley, 1995; Kerr et al., 1999; Gagnon et al., 2010). For instance, Bond and Midgley (1995) developed a “kill thy neighbor” model, which demonstrated that a trait promoting canopy flammability amongst a population of closely spaced conspecific individuals could increase reproductive fitness on the condition it also conferred other evolutionary advantages. Recently, Midgley (2013) has withdrawn his support for this model because of unrealistic assumptions, such as the need for the seed shadow of the flammable individual to closely align with the fire footprint, and for its seedlings that inherit the flammable trait to be more competitive in post-fire environments. Likewise, Midgley (2013) argues that the “pyrogenicity as protection” hypothesis (Gagnon et al., 2010), which posits that flammable crowns are protective of soil seed banks and subterranean bud banks, shares similar flaws to the Bond and Midgley (1995) model.

A feature of the discussion about the evolution of flammability is that flammability traits have been conflated with strategies that enable plants to recover following fire, such as resprouting from basal or aerial bud banks, and storing seeds in aerial or soil seed banks (Saura-Mas et al., 2010; Clarke et al., 2013). Such strategies manifestly increase the fitness of individual plants in fire prone landscapes. Traits that unambiguously assist post-fire recovery and regeneration can be used in ancestral trait reconstructions, illuminating evolutionary processes within clades. Examples include fire-cued flowering (Bytebier et al., 2011), the epicormic strands that allow eucalypts to resprout after fire (Crisp et al., 2011), and xylopodia and thick corky bark in South American savanna species (Simon et al., 2009) (Table 1). In contrast, traits that purportedly increase flammability are not so obviously related to the fitness of individuals. Some authors have rejected the notion that plants have evolved any traits to be flammable, indeed questioning the entire basis of the plant -fire evolutionary nexus (Bradshaw et al., 2011a). This leads to the basic question that is the subject of this review: “what plant traits and community attributes are known to increase flammability and could have arisen from natural selection through an evolutionary fire-feedback loop?” For the purposes of this review we define flammability as the propensity of living or dead plant material to ignite and sustain combustion.

## Flammability traits

### Biomass water content

**Figure 1:**
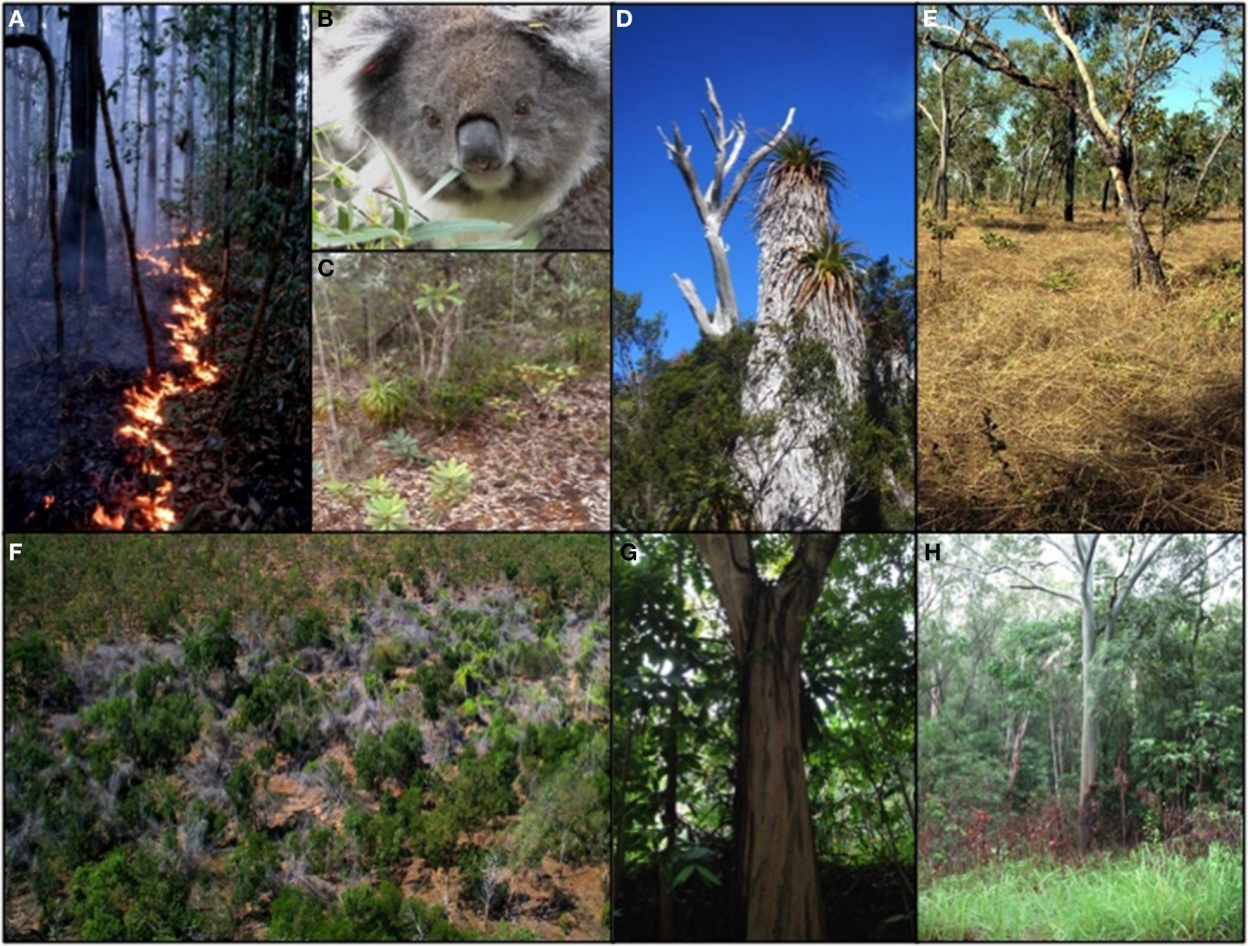
**Diverse plant traits that affect vegetation flammability. (A)** Surface fire in Amazonian rainforest leaf litter and ground cover vegetation during a severe drought, when leaf moisture context of living and dead foliage was very low (Photo: Mark Cochrane); **(B)** Koala (*Phascolarctos cinereus*), an iconic specialist mammalian herbivore involved in a co-evolutionary relationship with eucalypt leaf secondary chemical defenses. These defenses also make foliage exceptionally flammable (Photo Kath Handasyde); **(C)** New Caledonian maquis vegetation, which is dominated by sclerophyll species with phylogenetic links to Australian flammable heathland, yet has a poor capacity to recover from fire (Photo David Bowman); **(D)** leaf retention of *Richea pandanifolius*, a fire sensitive Gondwana rainforest giant heath, demonstrates that this trait is not universally associated with increasing flammability (Photo David Bowman); **(E)** low bulk density annual grass layer in eucalypt savanna is exceptionally flammable (Photo Don Franklin); **(F)** post-flowering die-off of the giant bamboo *Bambusa arnhemica* in frequently burnt eucalypt savanna. The dead bamboo is much less flammable than the grass layer in surrounding savanna (photo Don Franklin); **(G)** decorticating bark on a SE Asian tropical rainforest tree *Cratoxylum cochinchinense* demonstrates that this trait is not necessarily related to spreading fires via fire brands (Photo David Tng); **(H)** abrupt rain forest boundary in north Queensland which limits the spread of savanna fires, as evidenced by the shrubs burnt in the preceding dry season (Photo David Bowman).

Water in plant tissue is a heat sink, increasing the amount of energy required for fuels to ignite and sustain combustion. Therefore moisture content of living and dead fuels is the most fundamental constraint on biomass flammability (Gill and Moore, 1996; Alessio et al., 2008b; De Lillis et al., 2009; Alexander and Cruz, 2013; Murray et al., 2013) (Table 2). Leaf moisture content strongly affects flammability and is highly variable amongst life forms and biomes, exceeding 95% in succulents (Lamont and Lamont, 2000) and being as low as 20% in some sclerophyllous species (De Lillis et al., 2009). Although drought tolerating plants typically have more combustible living and dead foliage than mesic species, this correlation largely reflects the effect of the environment rather than inherent features that have evolved to increase flammability (Dickinson and Kirkpatrick, 1985; Berry et al., 2011; Hoffmann et al., 2012; Davies and Nafus, 2013; Seo and Choung, 2014). This point is exemplified by otherwise non-flammable rain forest foliage and litter beds burning under extreme drought conditions (Cochrane and Laurance, 2008) (Figure 1A).

**Table 2:** **Summary of the evidence for the effects and evolutionary origin of potential flammability**.

**Property**	**Evidence of effect on flammability**	**References**	**Evidence of evolution for flammability**	**References**
**LEAF**				
Leaf moisture content	Strong	Gill and Moore, 1996; Dimitrakopoulos and Papaioannou, 2001; Alessio et al., 2008a,b; De Lillis et al., 2009; Page et al., 2012; Alexander and Cruz, 2013; Murray et al., 2013	No	
Organic chemistry	Strong	Dickinson and Kirkpatrick, 1985; White, 1994; Owens et al., 1998; Kerr et al., 1999; Schwilk and Kerr, 2002; De Lillis et al., 2009; Holmes, 2009; Ormeno et al., 2009; Page et al., 2012; but see (Alessio et al., 2008a,b)	No	
Inorganic chemistry	Moderate	Dickinson and Kirkpatrick, 1985; Scarff and Westoby, 2006; Scarff et al., 2012	No	
Leaf dimensions	Moderate	Direct effect (Gill and Moore, 1996; Murray et al., 2013) and indirect effect through litter bed structure (Scarff and Westoby, 2006; Schwilk and Caprio, 2011; De Magalhães and Schwilka, 2012; Engber and Varner III, 2012)	No	
**WHOLE PLANT**				
Phenology	Strong	Bajocco et al., 2010; Ripley et al., 2010; Wittich, 2011; De Angelis et al., 2012	No	
Leaf retention	Moderate	He et al., 2011; Santana et al., 2011	Equivocal	He et al., 2011
Decorticating bark	Moderate	Ganteaume et al., 2009; Koo et al., 2010; Ellis, 2011	No	
Branch retention	Strong	Schwilk and Ackerly, 2001; Schwilk, 2003; Ne'eman et al., 2004; Keeley, 2012; Seo and Choung, 2014	Equivocal	He et al., 2012
Plant architecture	Moderate	Archibald and Bond, 2003; Schwilk, 2003; Mitsopoulos and Dimitrakopoulos, 2007; Hoffmann et al., 2012; Ledig et al., 2013	No	
**COMMUNITY**				
Fuel moisture	Strong	Dickinson and Kirkpatrick, 1985; Bowman and Wilson, 1988; Rollins et al., 2002; Ray et al., 2005; Jolly, 2007; Hoffmann et al., 2012; Alexander and Cruz, 2013; Davies and Nafus, 2013	No	
Fuel load	Strong	Rossiter et al., 2003; Brooks et al., 2004; Mitsopoulos and Dimitrakopoulos, 2007; Ganteaume et al., 2011; Hoffmann et al., 2012; McCaw et al., 2012; Scott et al., 2014; but see (Saura-Mas et al., 2010)	No	
Fuel arrangement	Strong	Bowman and Wilson, 1988; D'Antonio and Vitousek, 1992; Lippincott, 2000; Rollins et al., 2002; Archibald and Bond, 2003; Mitsopoulos and Dimitrakopoulos, 2007; Davies et al., 2009; Ganteaume et al., 2009, 2011; Berry et al., 2011; De Magalhães and Schwilk, 2012; Trauernicht et al., 2012; Van Altena et al., 2012; Castagneri et al., 2013; Davies and Nafus, 2013	No	
Canopy cover	Strong	Ray et al., 2005; Peterson and Reich, 2008; Warman and Moles, 2009; Hoffmann et al., 2012; Little et al., 2012; Murphy and Bowman, 2012; Trauernicht et al., 2012	No	

*Evidence for an effect on flammability is a necessary but not sufficient condition for demonstrating selection for flammability*.

### Organic chemistry

Foliar organic chemistry has a secondary effect on flammability after LMC (Alessio et al., 2008a,b; De Lillis et al., 2009; Page et al., 2012) (Table 2). For example, volatile organic compounds (VOCs such as terpenes and phenolics) can reduce ignition temperatures of living and dead leaves (Owens et al., 1998; Ormeno et al., 2009). However, VOCs also play an important role in herbivore defense (Owens et al., 1998; Page et al., 2012; Loreto et al., 2014), confounding their attribution as flammability adaptations (Dickinson and Kirkpatrick, 1985; Kerr et al., 1999; Schwilk and Kerr, 2002; Holmes, 2009). For example, variation in leaf terpenes of eucalypts, a notoriously flammable group of plants, is known to serve a wide variety of functions including influencing insect and mammalian herbivory and attracting insect pollinators, and has knock-on effects on decomposition and nutrient cycling (Keszei et al., 2008). Indeed, there is evidence of co-evolution between the diversification of plant secondary compounds and the intensity of special mammalian herbivores on eucalypt foliage (Moore et al., 2005) (Figure 1B).

### Inorganic chemistry

Leaves of flammable sclerophylls, which typically occur on infertile soils, have high foliar silica contents and low concentrations of other nutrients, especially phosphorus and nitrogen, compared to non-sclerophyll leaves (Turner, 1994). However, sclerophyllous foliage is imperfectly correlated with fire adapted vegetation (Midgley, 2013). The maquis shrublands of New Caledonia, for example, are dominated by sclerophyllous species, of which only about 19% persist through fires (McCoy et al., 1999) (Figure 1C), despite close phylogenetic links to fire-tolerant Australian heathland species. In principle, high phosphate concentrations in foliage could inhibit combustion given that phosphate is commonly used in fire retardants, yet little support has been found for this hypothesis (Scarff and Westoby, 2006; Scarff et al., 2012).

### Leaf dimensions

Leaf dimensions (size, thickness, and shape) influence the flammability of individual leaves. Thinner leaves, which have a high surface area to volume ratio and high specific leaf area, and larger leaves, appear to be more ignitable (Gill and Moore, 1996; Saura-Mas et al., 2010; Murray et al., 2013). However, species with small leaves tend to have narrow, frequently branched twigs and dense wood, which burn more intensely (Westoby and Wright, 2003; Pickup et al., 2005), potentially counteracting the lower flammability of small individual leaves. While flammability of live individual leaves may influence the spread of crown fires, surface fires are more strongly influenced by the flammability of litter beds. Large, long leaves may produce more flammable litter fuels because of lower packing density, which influences oxygen availability (Scarff and Westoby, 2006; Belcher et al., 2010; De Magalhães and Schwilk, 2012). For instance, an American study has found a link between abundance in litter fuels of *Pinus* species, which have long needle-shaped leaves, and fire severity (Schwilk and Caprio, 2011). Importantly, individual species have non-additive effects on litter flammability, which tends to be driven by the most flammable leaves in the litter (De Magalhães and Schwilk, 2012; Van Altena et al., 2012).

#### Dead leaf retention

When leaves die they are typically shed, although some plants retain dead leaves for extended periods; these dead leaves have low LMC relative to live foliage (Page et al., 2012). It has been suggested that dead leaf retention is an adaptation to promote plant flammability (He et al., 2011) and community flammability (Santana et al., 2011). He et al. (2011) used dated phylogenies to show that dead leaf retention in the Australian genus *Banksia* arose after the appearance of serotiny, suggesting that dead leaf retention could have evolved to increase the probability of fire and ensure that seeds are liberated. However, retention of dead foliage is not restricted to plants that occur in flammable environments: an example is the fire sensitive endemic Tasmanian rainforest arborescent monocot *Richea pandanifolia* (Figure 1D), signaling that this trait is not universally related to flammability. Indeed, it has been suggested that the retention of dead foliage in tall grasses is an adaptation to reduce the intensity of mammalian herbivory, but which may have also increased landscape fire activity (Mingo and Oesterheld, 2009; Antonelli et al., 2011).

### Phenology

In seasonally dry environments, phenology influences flammability by causing seasonal patterns in production and senescence of both leaves (deciduous plants) and of whole plants (annuals) (Keeley and Bond, 1999; Elliott et al., 2009; Bajocco et al., 2010; Ripley et al., 2010; De Angelis et al., 2012; Davies and Nafus, 2013). Obvious examples are senescence of annual herbs and grasses, leading to increased community flammability in the non-growing season because of high fine fuel loads (Wittich, 2011) (Figure 1E), as well as the dry season combustion of leaf litter in tropical dry forests (Mondal and Sukumar, 2014). This seasonal surge in available fuel has not been attributed to evolution, although Keeley and Bond (1999) hypothesized that synchronized mass flowering and die-off of bamboos is an evolutionary strategy to generate a “synchronous fuel load that significantly increases the potential for wildfire disturbance.” However, there is little evidence that fire is a key feature in the evolution of bamboo life-history (Saha and Howe, 2001). Franklin and Bowman (2003) found no support for this hypothesis from the north Australian giant bamboo, *Bambusa arnhemica*, which grows in an environment where fire is extremely frequent. The seedlings of this species did not require fire to establish, and dead adult biomass had low flammability (Franklin and Bowman, 2003) (Figure 1F).

### Decorticating bark

Lofted pieces of burning fuel (termed firebrands) can create spot fires ahead of a fire-front and are a key mechanism promoting fire spread (Koo et al., 2010). Decorticating eucalypt bark has been hypothesized to evolve to spread fires (Jackson, 1968; Mount, 1979). However, the individual fitness benefits of this trait are not obvious (Ellis, 1965). In any case decorticating bark also occurs in non-flammable environments (Figure 1G), and has been suggested as defending against epiphyte infestation (Carsten et al., 2002; Wyse and Burns, 2011).

### Self-pruning and branch retention

Shedding of dead lower branches reduces continuity between surface fuels and the canopy. Conversely, retained dead branches create fuel ladders and allow fires to reach the crown of individual trees and their neighbors (Schwilk, 2003; Keeley, 2012; Seo and Choung, 2014). Phylogenetic analysis shows that shedding of branches may have evolved in the genus *Pinus* to reduce crown fires (He et al., 2012), in contrast to the ancestral condition of branch retention that promotes crown fires. The latter is often associated with serotiny (Gauthier et al., 1996; Schwilk and Ackerly, 2001; Ne'eman et al., 2004), a derived trait that apparently offered an alternative strategy to deal with high fire activity during the Cretaceous (He et al., 2012).

### Plant architecture and canopy morphology

Plant architecture may also influence flammability. For instance, frequent fire on the New Jersey Pine Plains has selectively maintained a dwarf, crooked form of *Pinus rigida* which is more flammable than the surrounding tall forest (Ledig et al., 2013). In some Mediterranean environments, plants with fire-dependent seeding strategy have open crowns with fine leaves that promote flammability (Saura-Mas et al., 2010), although this crown morphology also occurs in environments where fire is not central to plant regeneration, such as South American shrublands with similar climates (Keeley et al., 2011a). Shading by dense canopies of individual trees influences understory floristics and local microclimate (Peterson and Reich, 2008; Cohn et al., 2011), thereby affecting fire regime. For example, closed crowned trees can suppress grasses in savannas (Hoffmann et al., 2012) (Figure 1H).

## Discussion

Our review has not been able to identify any individual plant traits attributes that exclusively influence flammability (Table 1). Further, we show that plant traits that increase flammability may exist in plant communities that are rarely burnt, suggesting they have evolved independently of landscape fire. It is probable that some traits related to flammability, such as foliar chemistry, may be “exaptations” (Gould and Vrba, 1982)—traits with another function that incidentally increases flammability (Trabaud, 1976; Snyder, 1984; Bradshaw et al., 2011a). Such micro-evolutionary processes are apparent in the selection of more flammable genotypes of *Ulex parviflorus* (Mediterranean gorse) (Pausas and Moreira, 2012; Moreira et al., 2014). The benefit of increased flammability for plants that require fire disturbance to regenerate is possibly greatest in environments where background fire frequency is low, for example in tall eucalypt forests compared to tropical eucalypt savannas (Bowman and Wilson, 1988; Murphy and Bowman, 2012). Increased flammability may also be of selective benefit for plants that recover following fire disturbance, thereby deflecting successional pathways from less flammable mature forests. For example, such a seral “niche construction” model has been proposed to explain the dynamics of eucalypt forests and rainforests in high rainfall areas of Australia (Jackson, 1968; Bowman, 2000). The eucalypt forests require fire to regenerate so that unless fire occurs within their life span the eucalypts are replaced by comparatively fire sensitive, continuously regenerating rainforest species (Tng et al., 2012). Clarke et al. (2014) tested this hypothesis and found that foliage and litter from eucalypt forest was not more flammable that from rainforest. Further, eucalypt forests regenerating after severe fire did not have more flammable litter compared to areas affected by less severe fire or long unburnt, so there was no evidence that fire selected for higher litter flammability. Likewise, Lindenmayer et al. (2011) have suggested that stands of *Eucalyptus regnans* regenerating following disturbance are inherently more flammable than long unburnt stands, yet a recent analysis shows this effect was not evident in stands burnt within the last 7 years, and was most pronounced in stands burnt around 15 years ago (Taylor et al., 2014), discounting the influence of short-lived herbaceous fire weeds that characterize the post-fire plant community (Jackson, 1968).

It is important to acknowledge that traits that influence plant combustion are not exclusively associated with flammability. This complicates macro-evolutionary ancestral state reconstructions by demanding joint consideration of the evolution of fire tolerating traits and recovery mechanisms with flammable traits. Mutch (1970) suggested that fire promoting traits followed the development of fire tolerating and recovery mechanisms, but it is possible that inherently flammable plants drove the evolution of plant recovery mechanisms—an evolutionary pathway known as “Mutch's converse” (Kerr et al., 1999; Schwilk and Ackerly, 2001; Schwilk and Kerr, 2002). The analysis of serotiny in *Banksia*, and self-pruning, bark thickness and serotiny in *Pinus* (e.g., He et al., 2011, 2012) suggest the latter, but many more ancestral trait reconstructions are required before generalizations can be drawn about the most typical evolutionary pathways, and how these patterns vary biogeographically. A confounding factor in such reconstruction is that plants that evolve traits to tolerate or recover from fire may be under less selection pressure to reduce their flammability, leading to positive correlations between flammability and fire tolerance without evolutionary selection for high flammability. Importantly, Midgley (2013) points out that selection for non-flammable traits, such as branch shedding, avoids many of the problems with the Mutch hypothesis, given the manifest individual fitness benefits of avoiding self-immolation. More research needs to be directed to this hypothesis, which we call “Midgley's alternative.”

The focus on flammability traits of individuals in both theoretical models and ancestral trait reconstructions obscures the fact that wildfire propagates through vegetation made up of multiple species, so the most appropriate unit of analysis should be the plant community. Community flammability is controlled by the interplay of climate with vegetation canopy cover, fuel continuity and litter bed characteristics (Table 1). This is well illustrated by boundaries between vegetation types with sharply contrasting flammability, such as savanna and tropical rainforests: forests which have closed canopies result in microclimates characterized by higher humidity, lower wind velocities, cooler temperatures, reduced evaporation and hence reduced fire risk compared to open-canopied savannas (Bowman and Wilson, 1988; Ray et al., 2005; Hoffmann et al., 2012; Little et al., 2012; Veldman et al., 2013). Litter beds are an emergent property of the plant community because the mix of dead foliage with different sizes and shapes affects fuel bulk density, which in turn influences flammability (Scarff and Westoby, 2006; Kane et al., 2008; Schwilk and Caprio, 2011; De Magalhães and Schwilk, 2012; Engber and Varner III, 2012; Van Altena et al., 2012; Murray et al., 2013; McGlone et al., 2014) (Figure 1H). Large, thin leaves and leaves with complex shapes (such as compound leaves or leaves with lobed margins) result in well aerated litter beds that typically dry out quickly and readily combust during dry periods (Scarff and Westoby, 2006; Kane et al., 2008; Schwilk and Caprio, 2011; De Magalhães and Schwilk, 2012; Engber and Varner III, 2012). The most extreme examples of this effect are tall tropical grasses, which produce highly combustible fuel beds, in contrast to denser leaf litter fuels: the difference in these fuel types reinforces forest-savanna boundaries (Hoffmann et al., 2012) (Figure 1H).

The stark differences in flammability of grasses and broadleaved fuels also invites consideration of the flammability traits amongst Poaceae lineages. Some grass genera have high flammability due to massive accumulation of fine, well-aerated fuels (e.g., *Andropogon*) (Setterfield et al., 2010), “haying-off” after the growing season (e.g., annual *Sorghum*) (Elliott et al., 2009), retention of dead foliage, or resin-rich leaves [e.g., *Triodia* (Allan and Southgate, 2002)]. Indeed, globally, many C4 savanna grasslands are maintained by fire (Scott et al., 2014). However, some other grasses are less flammable than surrounding vegetation, for example dense swards of Australian alpine *Poa* compared to surrounding heathlands (Williams et al., 2006). While invasive grasses can drive a grass fire cycle (D'Antonio and Vitousek, 1992; Setterfield et al., 2010), it is important to note that in many situations this feedback loop is driven by high anthropogenic ignitions and an absence of co-evolved grazers. More investigation of the flammable traits of grasses, and their evolutionary pathways, including co-evolutionary relationships with grazers (e.g., Linder and Rudall, 2005; Antonelli et al., 2011; McGlone et al., 2014) are warranted.

Clarke et al. (2014) used a mosaic of flammable eucalypt forest and far less flammable rainforest as an evolutionary “model system” to show there were no differences in the flammability of foliage of congeners in these contrasting forest types. They also found no differences in the flammability of litter fuels dried to a standard moisture content. This led them to reject the Mutch hypothesis that individual plant flammability is under natural selection; rather, they concluded that community flammability differences were related to the contrasting microclimates under the open eucalypt and the dense rainforest canopies. It is important to note that low flammability rainforest can establish beneath canopies of mature eucalypt forests growing in moist environments, blunting the view that eucalypt canopy openness is a specific adaptation to increase flammability (Tng et al., 2012).

Keeley et al. (2011b) argue that the most profitable route to disclosing the evolutionary relationships between plants and landscape fire is to understand the nexus between fire regimes and plant traits. However, we suspect fire regimes are too fluid to provide a sufficiently strong evolutionary pressure to select for highly flammable traits. Fire regimes respond rapidly to changing patterns of ignitions, intensity and type of herbivory, new species of invasive plants and longer term climate changes. For example, the loss of Pleistocene megafauna in both North America (Gill et al., 2009) and Australia (Rule et al., 2012) appeared to change fire regimes due to the proliferation of woody biomass, which fuelled more intense fires. Likewise, invasive species can abruptly change flammability by altering vertical or horizontal fuel continuity, and hence facilitate the spread of fires into canopies or amongst otherwise spatially isolated plants. This is well illustrated by the invasion of dry rainforests in Queensland by the woody shrub *Lantana camara*, which changes fire type from surface litter fires to shrub canopy fires that can kill rainforest trees, or invasive *Bromis tectorum*, which changes horizontal fuel continuity, causing loss of succulents such as giant saguaro cacti (*Carnegiea gigantea*) (Thomas and Goodson, 1992). Such shifting patterns of fire activity filtering numerous plant traits from multiple species make it difficult to sustain the notion that numerous species in communities have all evolved to collectively self-immolate. It is more parsimonious to view fire activity as a powerful filter that sorts plants with pre-existing flammabilities and hones regeneration strategies.

## Author contributions

David Bowman conceived the ideas for the manuscript, and Ben French carried out the initial literature review. All authors contributed to the writing.

### Conflict of interest statement

The authors declare that the research was conducted in the absence of any commercial or financial relationships that could be construed as a potential conflict of interest.
